# Predictors of short-term readmission after beyond total mesorectal excision for primary locally advanced and recurrent rectal cancer

**DOI:** 10.1007/s13304-019-00669-6

**Published:** 2019-06-27

**Authors:** Filomena Liccardo, Daniel L. H. Baird, Gianluca Pellino, Shahnawaz Rasheed, Christos Kontovounisios, Paris P. Tekkis

**Affiliations:** 1 Department of Colorectal Surgery, Royal Marsden Hospital, London, UK; 2 Department of Surgery and Cancer, Imperial College, 369 Fulham Rd, London, SW10 9NH UK; 3 Department of Advanced Medical and Surgical Sciences, Universitá della Campania “Luigi Vanvitelli, Naples, Italy; 4 Department of Colorectal Surgery, Chelsea and Westminster NHS Foundation Trust, London, UK

**Keywords:** Beyond TME, Pelvic exenteration, Rectal cancer, Complication, Readmission, Colorectal cancer

## Abstract

Unplanned readmissions heavily affect the cost of health care and are used as an indicator of performance. No clear data are available regarding beyond-total mesorectal excision (bTME) procedure. Aim of the study is to identify patient-related and surgery-related factors influencing the 30-day readmissions after bTME. Retrospective data were collected from 220 patients who underwent bTME procedures at single centre between 2006 and 2016. Patient-related and operative factors were assessed, including body mass index (BMI), age, gender, American Society of Anaesthesiologists’ (ASA) score, preoperative stage, neo-adjuvant therapy, primary tumour vs recurrence, the extent of surgery. The readmission rate was 8.18%. No statistically significant association was found with BMI, ASA score, length of stay and stay in the intensive care unit, primary vs recurrent tumour or blood transfusions. Not quite statistically significant was the association with pelvic side wall dissection (OR 3.32, *p* = 0.054). Statistically significant factors included preoperative stage > IIIb (OR: 4.77, *p* = 0.002), neo-adjuvant therapy (OR: 0.13, *p* = 0.0006), age over 65 years (OR: 5.96, *p* = 0.0005), any re-intervention during the first admission (OR: 7.4, *p* = 0.0001), and any post-operative complication (OR: 9.01, *p* = 0.004). The readmission rate after beyond-TME procedure is influenced by patient-related factors as well as post-operative morbidity.

**Electronic supplementary material:**

The online version of this article (10.1007/s13304-019-00669-6) contains supplementary material, which is available to authorized users.

## Introduction

Thirty-day readmissions after surgery are common and costly. Overall, unplanned readmissions after colorectal surgery have been estimated between 6 and 25%, while has been calculated that 11.0% of those are 30-day readmissions [1, 2]. The median cost for those readmissions has been estimated around $7030 (with a peak of $14,019) [2].

Among all patients with rectal cancer, 5–10% present a primary rectal cancer invading adjacent organs (beyond the total mesorectal excision plane LAPRC-bTME) and up to 10% develop local recurrence following primary surgery (LRRC) [3].

In those cases, R0 resection represents the strongest prognostic factor affecting long term survival [4–6]. Although R0 surgery requires complex procedures (exenterative or multivisceral resections), super specialist training of surgeons, the use of a variety of instruments to improve accuracy and surgical planning [7], the involvement of a multidisciplinary team and an attentive selection of the patients [8], it offers the best care option with 5-year survival rates up to 50% [3, 9].

The Pelvex Collaborative published the weight of different factors on long-term survival after bTME, and neoadjuvant therapy was associated with higher rates of readmission [10], but other variables were not taken into account. Aim of this study is to specifically investigate the role played by patient-related and surgery-related factors in the 30-day readmission rate after bTME procedures.

## Materials and method

### Data collection

The present manuscript adheres to the STROBE Checklist for Reporting of Observational Studies in Epidemiology (Suppl. Table 1).

A procedure-targeted database was created, including 220 patients who underwent bTME surgery for LAPRC and LRRC between May 2006 and November 2016 at the Royal Marsden Hospital. All patients were assessed by a multidisciplinary team, which decided the indications and the extent of surgery.

The following patient-related factors were assessed: body mass index (BMI), age, gender, American Society of Anaesthesiologists’ (ASA) score, preoperative stage, neo-adjuvant therapy, primary tumour vs recurrence.

With regards to the BMI, association with overweight (BMI > 25) and obesity (BMI > 30) was considered. Moreover, following the example of other studies [2], patients were cropped in two age groups: over and under 65 years of age.

The stage of the disease was assessed by CT and MRI scans plus PET scans, when deemed necessary, as per MDT decision (to confirm mass or identify occult metastases [3]) according to the TNM staging of colorectal carcinoma (AJCC 7th edition).

**Fig. 1:**
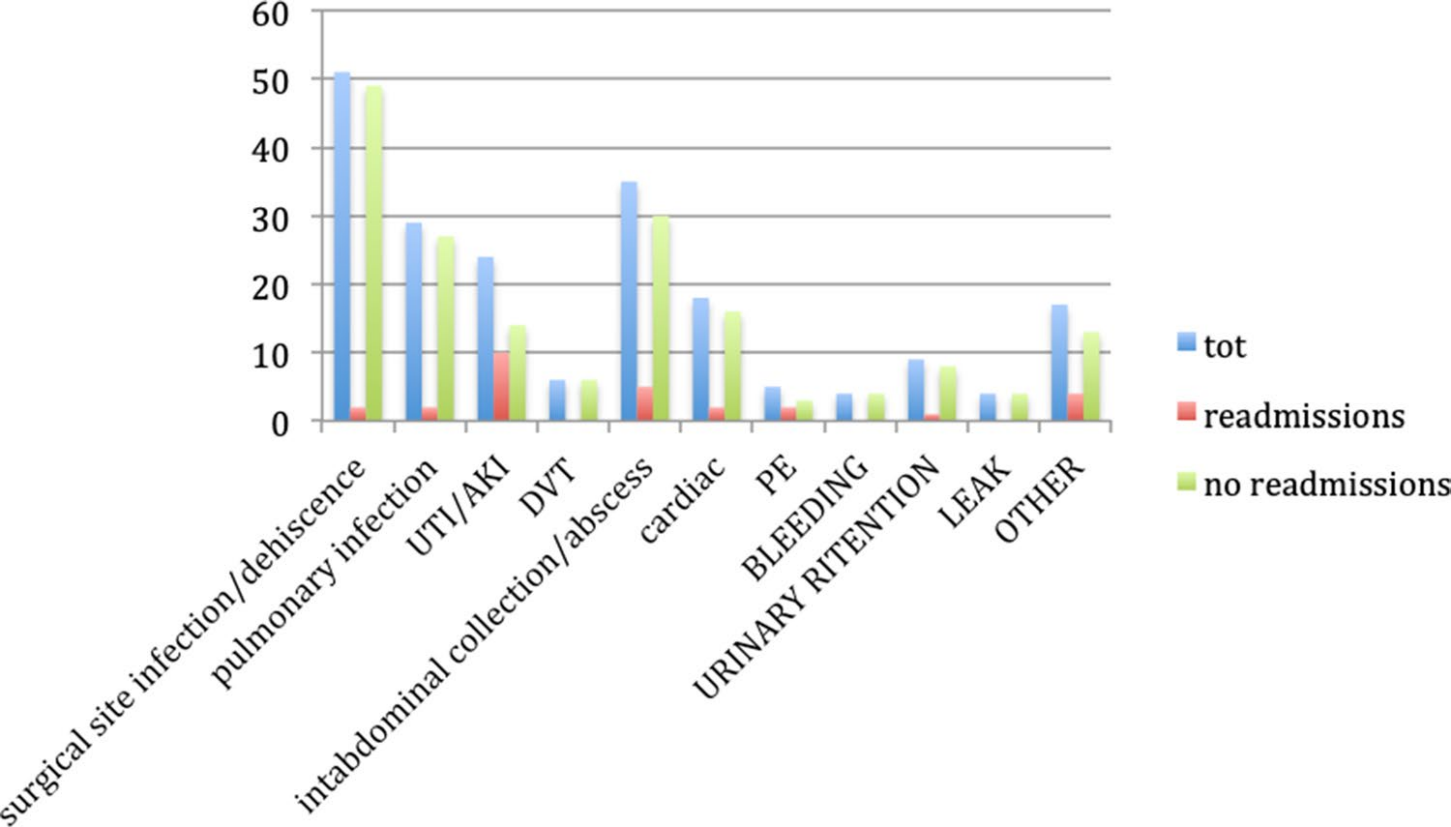
Postoperative complications. *DVT* deep vein thrombosis, *PE* pulmonary embolism, *UTI/AKI* urinary tract infection/acute kidney injury, *Tot* total

Operative and post-operative factors were also considered: plastic reconstruction technique, sacrectomy and pelvic side wall (PSW) dissection (as part of the procedure), blood transfusion at any time of the operation and during the recovery, length of stay (LOS), days spent in the intensive care unit (ICU), any re-interventions, and post-operative complications. The Clavien-Dindo (CD) classification was used to assess post-operative complications. With regards to re-interventions, both surgical and radiological ones were taken into account (Fig. 1).

### Definitions

LAPRC bTME: Locally advanced primary rectal cancer. This included patients with locally advanced primary rectal cancer bTME. These patients were identified by MRI, which predicted the need for an extended surgical resection beyond the TME plane to achieve an R0 resection
LRRC: Local rectal recurrence. Patients with recurrence, progression or development of new sites of tumour in the pelvis after previous resectional surgery for rectal cancer
PSW: Pelvic side wall
SBO: Small bowel obstruction

### Statistical analysis

Two-tailed Pearson Chi-squared test (when all expected cell frequencies were equal to or greater than 5) and Odds ratio (OR) with 95% confidence intervals (CI) were used for nominal variables, while Student’s *t* test was used for continuous variables, to examine the association of patient factors and intra-operative/post-operative factors with readmission. Independent contributors to readmission were determined at a significance level < 0.05.

## Results

Among the 220 patients there were no 30-day deaths, whereas the 30-day readmission rate was 8.18% (18 pts). Most of the readmissions were prompted by more than one cause, but urinary tract related complications registered the highest frequency (6/18—33.3%), while SBO (small bowel obstruction) prompted readmission in two cases (Table 1).

**Table 1:** Characteristics of the patients (*n* = 220)

Gender *n* (%)	
Male	138 (62.7)
Female	82 (37.3)
Age in years Mean ± SD (range)	61.7 ± 12.5 (28–89)
BMI in Kg/M^2^ Mean ± SD (range)	26.3 ± 4.3 (18.5–43)
ASA *n* (%)	
ASA I	18 (8.2)
ASA II	171 (77.7)
ASA III	31 (14.1)

### Association of patient-related factors with 30-day readmission

**Fig. 2:**
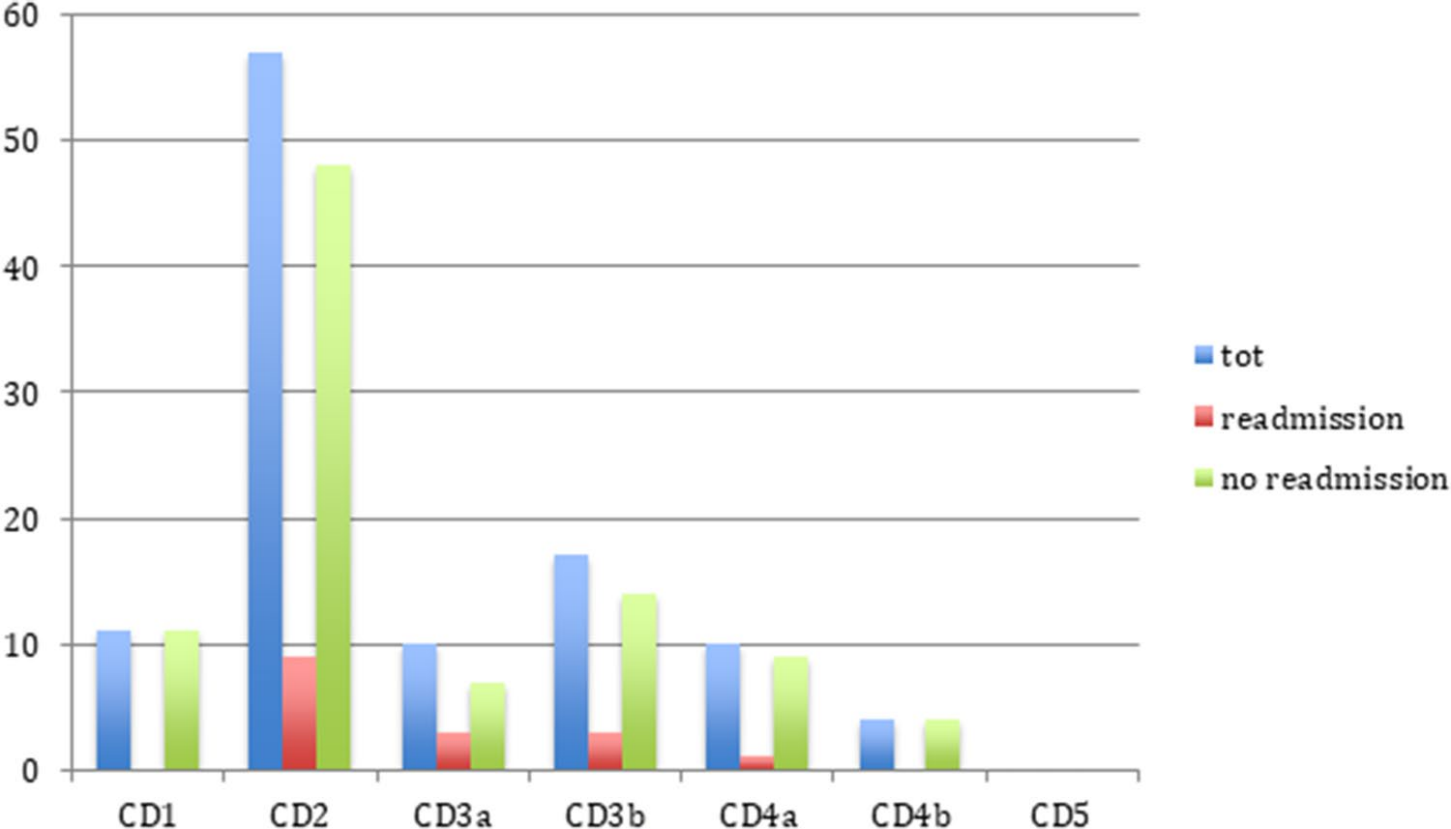
Postoperative complications according to Clavien-Dindo classification. *CD* Clavien-Dindo, *tot* total

Overall the mean age of the cohort was 61.5 ± 13.6 years, with a slightly higher value among the readmissions (63.7 ± 6.4). A statistically significant association with 30-day readmission (OR = 5.96; 95% CI: 2.19–16.18; *p* = 0.0005) was found in the over-65 group (Fig. 2).

Although the mean BMI was lower in the readmission group (25.8 vs 26.29 kg/m^2^), this was not statistically significant. There were no underweight patients (BMI < 18.50) and no association was found with a BMI > 25 (OR: 0.77; 95% CI = 0.29–2.03; Chi square Pearson = 0.28; *p* = 0.59). Only three patients in the readmission group had a BMI ≥ 30 (OR 0.77; 95% CI: 0.2147–2.8062; *p* = 0.6992).

The rate of male patients was higher in the readmission group (72.2%), but not statistically significant. With regards to ASA score, no statistically significant association among those with a score ≥ 3 was observed (OR: 0.77; 95% CI: 0.16–3.56; *p* = 0.74).

Out of 220 patients, 61 (27.7%) were diagnosed with a preoperative radiological IIIb stage or higher, while the percentage of patients falling in a post-operative histological staging of IIIb or higher was 25.45% (56/220). Of the 61 patients with a pre-operative stage of IIIb or higher, 11 were in the readmission group, showing a significant association (OR: 4.77; 95% CI: 1.75–12.98; *p* = 0.0022).

Although no association was found with patients operated on for recurrent rather than primary cancer (LRRC vs LAPRC), patients with recurrent disease were more common among those who were readmitted (respectively 61.1% of the readmissions vs 21.3% in the no-readmissions group).

The large majority of the readmitted patients underwent neo-adjuvant chemo-radio therapy: 91.4% out of the total and 66.7% of the readmissions (12/18). The association with 30-day readmission was statistically significant (OR = 0.13; 95% CI: 0.044–0.425; *p* = 0.0006).

Results are summarized in Table 2

**Table 2:** Association of patient-related factors with 30-day readmission

	Overall	Readmission	No readmission	Results
Age	61.5 (13.6)	63.7 (6.4)	61.5 (13.2)	*p* = 0.48
Age > 65 year	45 (99/220)	55.5 (10/18)	17.3 (35/202)	*p* = 0.0005
BMI	26.29 (4.92)	25.80 (4.19)	26.39 (4.52)	*p* = 0.59
BMI > 25 kg/m^2^	18/220 (8.2%)	10/18 (55.5%)	125/202 (61.9%)	*p* = 0.59
BMI ≥ 30 kg/m^2^	42/220 (19.09%)	3/18 (16.6%)	39/202 (19.3%)	*p* = 0,69
Male gender	138/220 (62.7%)	13/18 (72.2%)	125/202 (61.9%)	*p* = 0.76
ASA ≥ 3	30/220 (13.6%)	2/18 (11.1%)	28/202 (13.9%)	*p* = 0.74
LRRC	54/220 (24.5%)	11/18 (61.1%)	43/202 (21.3%)	*p* = 0.09
STAGE ≥ IIIb	61/220 (27.7%)	11/18 (61.1%)	50/202 (24.75%)	*p* = 0.0022
Neoadjuvant therapy	201/220(91.4%)	12/18 (66.7%)	189/202 (93.6%)	*p* = 0.0006

Results are mean (standard deviation) or *n* (%)

*ASA* American Society of Anaesthesiologists’ score, *BMI* body mass index, *LRRC* locally recurrent rectal cancer

### Association of operative and post-operative factors with readmission

One hundred and six patients underwent a flap procedure (48.2%): 60 (56.6%) an oblique rectus abdominis myocutaneous flap (ORAM), 28 (26.4%) a vertical rectus abdominis myocutaneous flap (VRAM), 15 (14.1%) gluteal, and 3 (2.8%) a gracilis muscle flap. While the rate of flaps procedures among the readmissions was slightly lower (44.4%) when compared to the no-readmission group (48.5%), this difference was not statistically significant (OR: 0.84; 95% CI: 0.32–2.23; Pearson Chi square: 0.11; *p* = 0.74).

Low sacrectomy (S4–S5) and PSW (pelvic side wall) dissection were performed as part of the operation in 34 (15.4%) and 20 (9%) patients. Sacrectomy was not associated with the readmissions (OR: 2.29; 95% CI: 0.76–6.91; *p* = 0.14), nor was PSW dissection (OR: 3.32; 95% CI: 0.97–11.28 *p* = 0.054, Fisher’s exact test 0.066).

Overall, 41.8% of patients needed at least one unit of blood transfusion during the first admission, but no significant difference was noted between those who were readmitted or not (OR: 0.87; 95%: 0.32–2.35; *p* 0.79).

The mean of days spent in ICU was 4.26 ± 6, while the mean LOS in the hospital was 18.14 ± 11.49 days. Both values were higher in the readmission group (4.67 and 20.8, respectively), but again this difference was not statistically significant.

The majority of post-operative complications fell under CD2 in both the groups, while no CD5 complications were encountered. While wound or flap infection/dehiscence were the most frequent complications during the first admission in the entire cohort (51/220; 23.2%), when considering only the readmission group, urinary tract infection and acute kidney injury occurred in 55.5% of patients (10/18; *p* = 0.0001).

A strong association was also found between the presence of any morbidity during the first admission and the readmissions (OR: 9.01; 95% CI: 2.01–40.21; *p* = 0.004; Pearson Chi-squared test 11.58; *p* = 0.0007).

The number of re-interventions during the first recovery was significantly higher in the readmission group (OR: 7.4167; 95% CI: 2.6811–20.5168; *p* = 0.0001; Fisher exact test: *p* = 0.0002), even though, the proportion of surgical and radiological re-interventions seemed comparable among the two groups.

Table 3 depicts the findings.

**Table 3:** Association of surgery-related factors with 30-day readmission

	Overall	Readmission	No readmission-	Results
Flap	106/220 (48.2%)	8/18 (44.4%)	98/202 (48.51%)	*p* = 0.74
Sacrectomy	34/220 (15.4%)	5/18 (27.8%)	29/202 (14.3%)	*p* = 0.14
PSW	20/220 (9%)	4/18 (22.2%)	16/202 (7.9%)	*p* = 0.054
Transfusions	92/220 (41.8%)	7/18 (38.9%)	85/202 (42%)	*p* = O.7
ICU stay, days	4.26 (6)	4.67 (3.16)	4.23 (6.21)	*p* = 0.76
LOS, days	18.14 (11.49)	20.78 (11.66)	17.93 (11.53)	*p* = 0.6
Reintervention	33/220 (15%)	9/18 (50%)	24/202 (11.8%)	*p* = 0.0001
Surgical	11/33 (33.3%)	3/9 (33.3%)	8/24 (33.3%)	*p* = 0.03
Radiological	22/33 (66.7%)	6/9 (66.6%)	16/24 (66.6%)	*p* = 0.001
Post-operative morbidity	111/220 (50.5%)	16/18 (88.9%)	95/202 (47%)	*p* = 0.004

Results are mean (standard deviation) or *n* (%)

*ICU* intensive care unit, *LOS* length of stay, *PSW* pelvic side wall dissection

## Discussion

The costs of unplanned readmissions after colorectal surgery are high [2], and bTME surgery itself requires a large amount of resources and high level of expertise [3]. Therefore, predicting and preventing the causes of readmission after this type of surgery is crucial.

When considering patient-related factors, the impact of the age on the risk of 30-day readmission is still matter of debate after colorectal surgery. While some authors found a significant association [11], this was not confirmed by other studies [12]. Biss et al. [2] even found age < 65 years to be one of the patient factors associated with an increased risk of readmission after colonic and rectal resections, while in our study a statistically significant association with 30-day readmissions (OR = 5.96; 95% CI: 2.19–16.18; *p* = 0.0005) was found in the over-65 group.

This may be due to the fact that Bliss et al. considered also surgery for inflammatory bowel diseases (IBD) and non-elective surgery. In fact, the authors themselves suggested that a higher acuity of illness in the younger IBD patients, along with aversion to decline operation on sicker young patients, might represent a possible explanation to this discrepancy.

Neoadjuvant therapy has been associated with a higher risk of readmission [2]. Similarly, IIIb stage disease or higher and neo-adjuvant chemo-radio therapy were found significantly associated to unplanned readmissions in our study. Clearly, patients with higher stage disease are more prone to undergo neo-adjuvant therapy, suggesting the presence of a link, which might be worth investigating.

The impact of higher BMI on the postoperative events is still matter of debate: the study conducted by the EuroSurg collaborative in 2018 on 2519 patients across 127 centres undergoing major gastrointestinal surgery, found different outcomes depending on whether patients were undergoing surgery for benign or malignant disease. In fact, the individual patient meta-analysis demonstrated that obese patients undergoing surgery for malignancy were at increased risk of major complications (OR 2.10, 95% CI 1.49–2.96, *p* < 0.001), whereas obese patients undergoing surgery for benign indications were at decreased risk (OR 0.59, 95% CI 0.46–0.75, *p* < 0.001) compared to normal weight patients [13]. With regards to Beyond TME Surgery, Baird et al. found no significant difference between the three groups of patients with normal weight (BMI 18.5–24.9), overweight (BMI 25–29.9) and obese (BMI ≥ 30), in terms of postoperative morbidity or overall survival [14].

On the other hand, high BMI has been elsewhere identified as one of the prognostic factors for readmissions. A multivariate analysis conducted by Poelemeijer et al. [15] identified BMI ≥ 30 kg/m^2^ as an independent predictor of a complicated postoperative course after colorectal cancer surgery, and to be associated with a higher readmission rate. Despite that, no association was found with a BMI > 25 (OR: 0.77; 95% CI = 0.29–2.03; Chi square Pearson = 0.28; *p* = 0.59) or a BMI ≥ 30 (OR 0.77; 95% CI: 0.2147–2.8062; *p* = 0.6992) among our patients.

Pucciarelli et al. [16] found that male gender increased the risk of 30-day readmission after surgery for primary colorectal cancer. In the present study, the rate of male patients was higher in the readmission group (72.2%), but not statistically significant.

With regards to ASA, Bennedsen et al. [17] found that an ASA score ≥ 3 was significantly associated with 30-day readmission in an enhanced recovery after surgery cohort undergoing colorectal surgery, while our data showed no statistically significant association.

When considering the operative factors, en bloc sacrectomy (regardless of tumor histology) has been associated with a high rate of major complications often necessitating readmissions and secondary interventions [18]. In our study, 34 low sacrectomies were performed and were not significantly associated with the readmissions (OR: 2.29; 95% CI: 0.76–6.91; *p* = 0.14).

Moreover, Rencuzogullari et al. [19] investigated the role of the use of muscle flap in complex surgery, and not only found an association with higher risk of wound dehiscence, but also demonstrated that patients with wound dehiscence had a higher rate of readmission, need for reoperation and an increased risk of 30-day mortality. Nonetheless, while the rate of flaps procedures among the readmissions was slightly lower (44.4%) in our study, this difference was not statistically significant (OR: 0.84; 95% CI: 0.32–2.23; Pearson Chi square: 0.11; *p* = 0.74).

With regards to the post-operative factors, in our study the mean LOS was higher in the readmission group, but this difference was not statistically significant. On the other hand, other authors have demonstrated the impact of LOS on the unplanned readmissions: Kelly et al. [20] found that a postoperative LOS ≥ 8 days after colorectal surgery was associated with a 55% increase in the relative hazard of readmission; and a prolonged LOS was found to be independently associated with an increased likelihood of readmission (OR, 1.42; 95% CI 1.32–1.52) by Zafar et al. [21]. Arguably, this can be related to the presence of complications during the post-operative period, which prolonged the recovery.

Post-operative morbidity and the need for reinterventions, in fact, affected the risk of readmission after colorectal surgery in several studies [11, 22, 23]. Similarly, amongst our patients a strong association was found between the risk of 30-day readmission and the presence of any morbidity during the first recovery as well as the need for re-interventions. When considering beyond-TME surgery, the morbidity has been previously reported between 42 and 60% [3, 24, 25]. Consistently, in the present study the morbidity reached a rate of 50.5%. Nevertheless, the rate falls to 18.6% (41/220) when taking into account only complications classified as CD3 or over. In fact, the majority of post-operative complications fell under CD2 in both readmission and no-readmission groups, while no CD5 complications were encountered. Moreover, consistently with our study, Bliss et al. [2] found that patients who received neoadjuvant therapy were not only more likely to be readmitted, but had also more postoperative complications (unadjusted OR 1.53), and radiological reinterventions (unadjusted OR 2.12), suggesting a possible link between those features.

Keller et al. [26] found ICU stay to be a predictive factor for readmission after colorectal surgery (*p* = 0.021; OR 3.16; 95% CI 1.19–8.39). Nevertheless, our study showed no significant difference between the readmission and no-readmission group (*p* = 0.76).

Other factors which had been associated with 30-day readmission include high estimated blood loss [18], low Hb and need for transfusions [27]. In the current study, despite the fact that the 41.8% of patients needed at least one unit of blood transfusion perioperatively, no significant association was found (*p* = 0.79).

Limitations of the current study include its retrospective fashion and the fact that it was performed in a single centre. However, it was conducted in a referral centre for bTME surgery, and it is the first to assess the rate of readmission in a structured way. It has been suggested that high degree of variability of reporting outcomes exists in patients undergoing surgery for LAPRC and LRRC [5]. Available guidelines on colon and rectal cancer do not specifically recommend collecting this parameter [28, 29]. Thirty-day readmission might be an important quality indicator, worth including consistently within a standardization of reporting on the outcomes of bTME among other variables that need to be considered in patients with advanced colorectal cancers [5, 30, 31].

## Conclusion

To reduce the costs of bTME surgery, predicting and preventing the causes of readmissions after this type of surgery is crucial. Patients characteristics (age, stage), pre-operative treatments (neoadjuvant therapy) and postoperative morbidity, appear to influence the risk of readmission more than the specific surgical procedures performed.

There were some discrepancies among some of the previous publications, and between previously published results and those of the current study, including the influence on readmission of age [2, 11, 12], BMI [13–15], gender [16], ASA score [17] or LOS [20, 21]. Randomised studies on beyond-TME surgery should address this aspect in details.

Our results show that morbidity following this type of surgery is still high. In particular, the rate of UTI/AKI was 10.9%, and in line with the literature [32], probably due to the obvious strict anatomical rapport between rectal cancer and urinary tract. Postoperative UTI are associated with longer LOS, higher reoperation rate, higher 30-day mortality and other complications [33]. These findings suggest the need for protocols preventing not only post-operative morbidity in general, but specifically urinary tract related complications.

## Electronic supplementary material

Below is the link to the electronic supplementary material.

Supplementary material 1 (DOC 88 kb)
